# Differentiation between acute macular neuroretinopathy and paracentral acute middle maculopathy in elderly persons: two case reports

**DOI:** 10.1186/s12886-021-02218-5

**Published:** 2021-12-27

**Authors:** Qin Zhang, Xiuhong Qin, Ling Xu

**Affiliations:** 1 Shenyang He Eye Specialist Hospital, No. 128 Huanghe North Street, Shenyang, 110034 Liaoning Province China; 2 Department of Ophthalmology, First Affiliated Hospital of Dalian Medical University, No. 222 Zhongshan Road, Xigang Strict, Dalian, 116011 Liaoning Province China

**Keywords:** Acute macular neuroretinopathy, AMN, Paracentral acute middle maculopathy, PAMM, Optical coherence tomography, Optical coherence tomography angiography

## Abstract

**Background:**

We report one case of rare acute macular neuroretinopathy (AMN) in an elderly patient with hypertension and one case of common paracentral acute middle maculopathy (PAMM) in a patient with diabetes mellitus to illustrate the difference between the two diseases.

**Case presentation:**

This report describes two cases, one involving AMN and the other PAMM. The first patient was a 70-year-old man complaining of blurred vision for 3 days. He was examined with fundus photography, optical coherence tomography angiography (OCTA) and optical coherence tomography (OCT); a diagnosis of AMN was established. The second patient was a 50-year-old woman who complained of decreased vision during the past month. She had had diabetes mellitus for 6 years. From the ophthalmic imaging examination, the patient was diagnosed with PAMM and non-proliferative diabetic retinopathy (NPDR). Both patients were treated with drugs for improving microcirculation and neurotrophic drugs; however, there was no significant improvement in visual acuity.

**Conclusions:**

AMN is more common in young patients and is rarely observed in elderly patients with systemic diseases. The OCTA examination has an auxiliary diagnostic value for deep retinal capillary network ischaemia. Meanwhile, OCT examination has important imaging value in differentiating AMN from PAMM and can help avoid missed diagnoses.

## Background

Acute macular neuroretinopathy (AMN) was first reported by Bos and Deutman in 1975. It is a rare form of macular degeneration of unknown pathogenesis [1]. It often occurs in young women, and it can be manifested as sudden single or multiple paracentral scotomata in one or both eyes. The fundus presents as one or more flat wedge-shaped lesions in the macula [2]. With the development of detection technology, optical coherence tomography (OCT) has become an important means to further understand AMN. It has been found that AMN presents as macular outer layer retinopathy, that is, ellipsoid zone and outer nuclear layer missing. David Sarraf classified AMN into type I paracentral acute middle maculopathy (PAMM) and type 2 AMN according to the location of the lesion on OCT [3]. The incidence of AMN is low and clinically rare, and most previous cases were reported in young patients. Here, we report a rare case of AMN in an elderly patient with hypertension. We also report a case of diabetes mellitus complicated with PAMM in a woman to analyse the difference between them.

## Case presentation

### Case 1

**Fig. 1:**
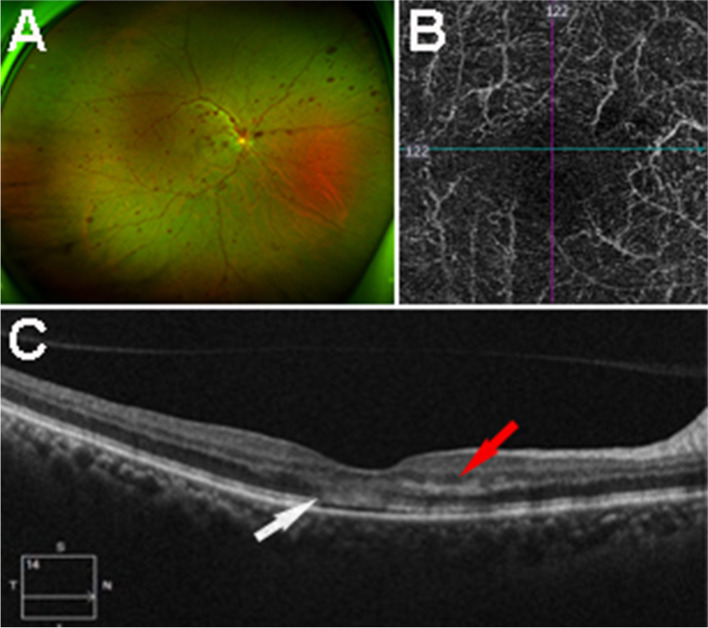
**A** Fundus photograph revealed vasodilatation of the vein and numerous patchy haemorrhages in the retina of the right eye. **B** Optical coherence tomography angiography (OCTA) showed a significant decrease in the density of the deep capillary in the right eye. **C** OCT examination revealed a hyperreflective plaque-like lesion in the outer plexiform layer (red arrow), involving the ellipsoid zone and the interdigitation zone at the centre of the macula (white arrow)

A 70-year-old male patient came to our department on 28 May 2020 due to a sudden loss of vision in his right eye. He had been suffering from high blood pressure (BP) for more than 10 years and taking antihypertensive medication. The patient’s BP was 170/90 mmHg. No other history of systemic disease or family history was recorded. Ophthalmologic examination revealed a best corrected vision acuity (BCVA) of FC/30 cm OD and 0.8 OS. Intraocular pressure (IOP) was 17 OD and 19 OS. There was only mild opacity in the lens cortex in the anterior segment in both eyes. Fundus examination of the right eye showed a clear margin of the optic disc, cup-to-disc ratio (C/D) of 0.3, tortuosity and vasodilatation of the vein, thin arteries and cross-compression signs. In addition, numerous round, linear and patchy haemorrhages in the posterior pole and midperiphery of the retina were observed, and absence of normal foveal reflection (Fig. 1A). Fundus examination of the left eye revealed that it was normal. Optical coherence tomography angiography (OCTA) showed a significant decrease in the density of the deep capillary in the right eye (Fig. 1B) and no abnormal OCTA in the left eye.

OCT revealed a hyperreflective plaque-like lesion in the outer plexiform layer in the macula region of the right eye, involving the ellipsoid zone and the interdigitation zone at the centre of the macula. The retinal pigment epithelium was intact (Fig. 1C). Carotid artery Doppler (CAD) ultrasound showed several plaques in the internal carotid artery. The patient was diagnosed with AMN and non-ischaemic central retinal vein occlusion (CRVO) in the right eye and early cataract in both eyes. He was treated with microcirculatory and neurotrophic drugs. After 2 months of follow-up, the vision acuity was 0.01 OD and 0.8 OS. There was no apparent improvement in vision in the right eye.

### Case 2

The second patient was a 50-year-old female. She came to our department on 17 June 2020 because of the loss of binocular vision for a month. She had had diabetes for 6 years and hypertension for 2 years. Her blood sugar levels and BP were stable under treatment with hypoglycaemic and antihypertensive drugs. No other history of systemic disease or family history was apparent. The BCVA was 0.5 OD and 0.4 OS. The IOP was 18 mmHg OD and 17 mmHg OS. There was only mild opacity in the lens cortex in both eyes in the anterior segment.

**Fig. 2:**
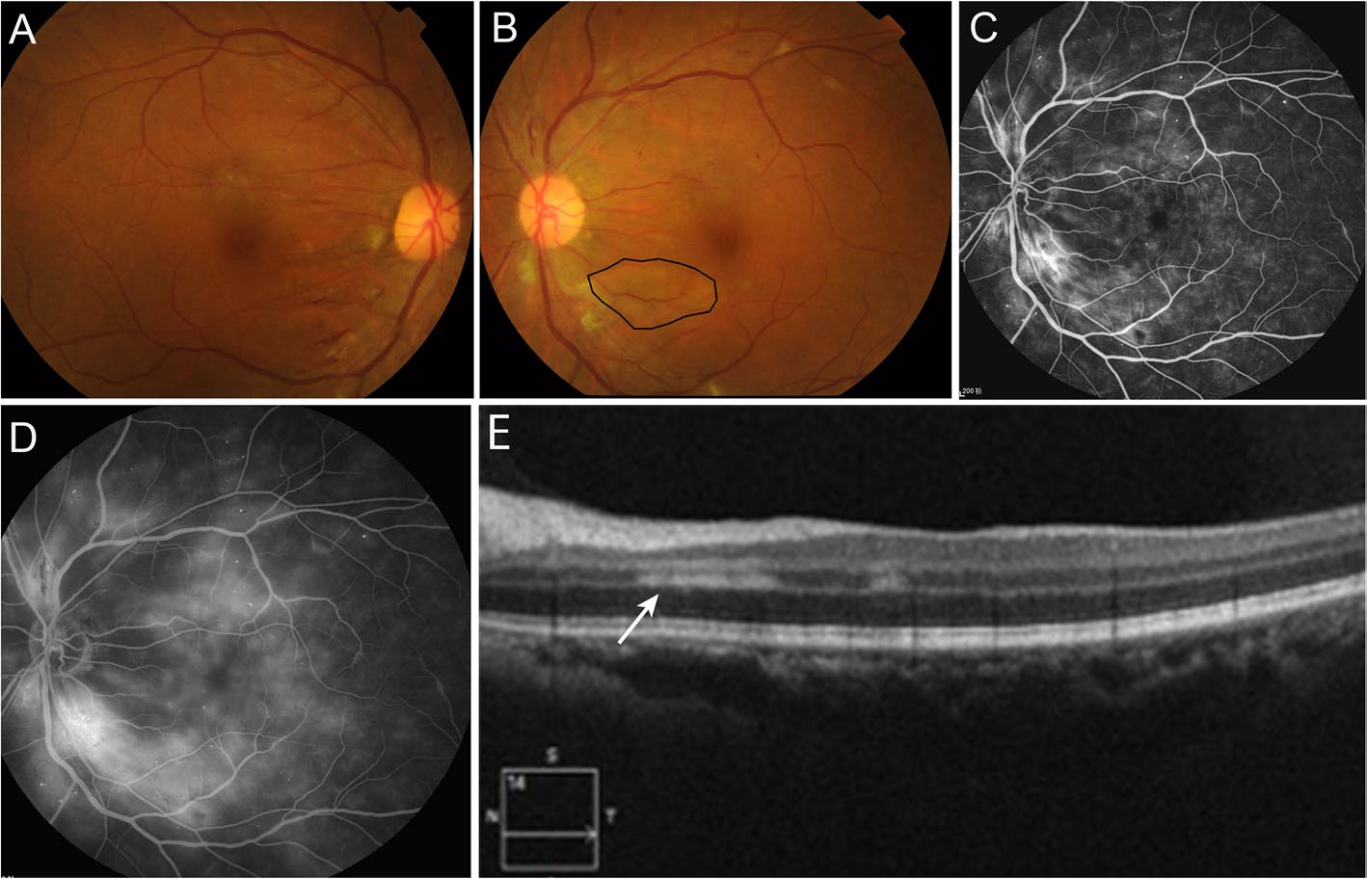
**A** Fundus photograph revealed microaneurysm, haemorrhage and cotton wool scattered in the retina in the right eye. **B** The fundus of the right eye is similar to that of the left eye except the grey-white lesion (black circle) below the macula. **C** Fundus fluorescein angiography (FFA) revealed multiple hyperfluorescence spots in the posterior pole and hypofluorescence lesion in the cotton wool in the left eye. **D** Leakage of fluorescein in the macular at late stage in the left eye. **E** Optical coherence tomography (OCT) examination in the left eye suggested high reflex bands in the inner plexiform layer and inner nuclear layer (white arrow)

Fundus examination revealed a normal optic disc with microaneurysm and haemorrhage scattered in the posterior pole retina in the left eye. Cotton-wool spots seen around the optic disc, and a wedge-shaped grey-white lesion was observed below the macula (Fig. 2B). The fundus of the right eye was similar to that of the left eye, besides there was no grey-white lesion below the macula in the right eye (Fig. 2A). Fundus fluorescein angiography (FFA) revealed multiple hyperfluorescence spots in the posterior pole and hypofluorescence lesion in the cotton wool (Fig. 2C), as well as late-stage leakage in the macular (Fig. 2D) in the left eye. The FFA of the right eye was similar to that of the left eye. OCT examination in the left eye showed the high reflex bands in the inner plexiform layer and inner nuclear layer, which did not involve the outer retina (Fig. 2E).

No abnormality was found in the right eye by OCT. The patient was diagnosed with PAMM in the left eye, non-proliferative diabetic retinopathy (NPDR) bilaterally and early cataract in both eyes. Blood glucose and BP were controlled, and microcirculatory and neurotrophic drugs were given for the treatment. At 2 months of follow-up, the BCVA was 0.5 OD and 0.8 OS, with no apparent improvement.

## Discussion and conclusions

AMN is a rare ocular fundus disease in young women; the common causes are oral contraceptives, coffee intake, epinephrine or ephedrine use, trauma, migraine, viral infection, postpartum hypotension and so on [2]. Sarraf et al. divided AMN into two types: Type I is called PAMM, where the lesion is located in the inner nuclear layer; type II is called AMN, where the lesion was located in the outer nuclear layer [3]. The occurrence of PAMM is due to deep retinal capillary ischaemia. It is characterised by a high reflection band in the inner nuclear layers and the junction of inner and outer plexiform layers in the acute phase and atrophy and thinning of the inner nuclear layer in the chronic phase, observed through OCT examination. In contrast, AMN is characterised by a strong reflection band of the outer nuclear layer involving the ellipsoid zone and the interdigitation zone in the acute phase with the outer nuclear layer thinned in the chronic phase [4]. The fundus appearance of both diseases is similar. PAMM is commonly seen in the elderly, often secondary to retinal arteriovenous occlusion, diabetic retinopathy, ocular ischaemia syndrome, sickle cell disease and other systemic diseases [5].

The first patient was an old man. In his right eye, the manifestation of a strong reflection band in the outer nuclear layer involving the ellipsoid zone and the interdigitation zone in OCT, and the significantly decreased density of the deep retinal capillary in OCTA [6] was consistent with AMN. However, this patient was older and had CRVO in his right eye, with a history of hypertension, which was different from previous reported cases. In 2019, Wyss et al. [7] reported four cases, all young women, involving AMN with focal abnormalities in the photoreceptor outer segments. In the same year, Imran Ashfaq et al. [8] also reported four cases of AMN aged 18–32 years associated with confirmed influenza virus infection. In 2020, Alessandro Porta et al. [9] reported a bilateral AMN in a young female patient without any risk factors and different progression rates in the two eyes. In 2021, Rony C Preti et al. [10] described a case of AMN in a 70-year-old man with sudden visual loss of Covid-19 infection. And in 2021, Mariana Nadais Aidar et al. [11] reported a 71-year-old woman complaining of low visual acuity diagnosed of AMN 14 days after the COVID-19 infection.

Sarraf et al. [3] believes that the direct cause of AMN is retinal surface or deep capillary obstruction. We inferred that the pathology of AMN in our case may be related to the intracellular oedema induced by deep capillary ischaemia and hypoxia in CRVO, which ultimately led to oedema of the outer nuclear layer, ellipsoid zone and interdigitation zone. CRVO leads to a decrease in whole retinal blood flow followed by reduce in blood supply of macular, finally leading to deep capillary ischaemia in the macular area. After 2 months’ follow-up with therapy, only partial haemorrhage was absorbed. No significant changes were found in the outer nuclear layer, ellipsoid zone or interdigitation zone.

The second patient was a middle-aged female with a history of diabetes mellitus who had bilateral diabetic retinopathy. The OCT examination showed oedema of the inner plexiform layer and inner nuclear layer in the paramacular in the left eye without outer retina involvement, which was consistent with PAMM. Diabetic retinopathy is thought to be a contributing factor in this case. It is considered that the pathogenesis of PAMM is related to the oedema of cells in the inner nuclear layer caused by intermediate and deep retina capillary ischaemia. Moreover, the perimacular retina is thick with high oxygen consumption, therefore, diseases that cause abnormal blood supply to the retinal capillary network in macular area can cause PAMM. In addition, FFA mainly displays the vascular network on the surface of retina, and the middle and deep retinal blood flow can’t be accurately displayed. So, PAMM often has no abnormal performance in FFA. And there was no effective treatment for AMN and PAMM at present. The efficacy of systemic steroids therapy was not clear.

Both patients were middle-aged and elderly with hypertension or diabetes. One patient developed PAMM, while the other developed AMN. AMN has multiple aetiologies [12]; yet, there are few reports of AMN in the elderly. AMN mainly occurs in young women. In our first case, it occurred in CRVO secondary to hypertension in an elderly patient, which was a rare presentation. It is speculated that deep capillary ischaemia caused by CRVO is the pathogenetic factor of AMN; therefore, although elderly patients with systemic diseases are prone to develop PAMM, AMN may also arise, and this should be emphasised by ophthalmologists.

## Data Availability

All data generated or analysed during this study are included in this published article.

## Abbreviations

AMN: Acute macular neuroretinopathy
PAMM: Paracentral acute middle maculopathy
OCT: Optical coherence tomography
OCTA: Optical coherence tomography angiography
FFA: Fundus fluorescein angiography
NPDR: Non-proliferative diabetic retinopathy
CRVO: Central retinal vein occlusion
